# CD44variant exon 9 plays an important role in colon cancer initiating cells

**DOI:** 10.18632/oncotarget.1048

**Published:** 2013-06-06

**Authors:** Youhei Kimura, Takanori Goi, Toshiyuki Nakazawa, Yasuo Hirono, Kanji Katayama, Takeshi Urano, Akio Yamaguchi

**Affiliations:** ^1^ First Department of Surgery, University of Fukui, Fukui, Japan; ^2^ Department of Biochemistry, Shimane University School of Medicine, Izumo, Japan, Japan

**Keywords:** colon cancer, CD44 variant exon, stem cells, Cancer initiating cells

## Abstract

Cancer stem cells(cancer initiating cells) have become increasingly important in the treatment of malignant tumors. CD44 in particular has been identified as a marker for stem cells in colon cancer, which is a high-morbidity tumor. However, many details remain unknown, including identification of the relevant exon. The elucidation of these details could lead to the development of new therapies and improvements in prognosis. We report our findings on the importance of CD44 variant exon 9(v9) of stem cells in colon cancer.

Using the anti-CD44 standard form(s) antibody, as well as antibodies for each of the CD44 variant exons, we studied colon cancer cell lines by examining stained images of stem cells in the crypt of normal colon mucosa. Using the anti-CD44v9 antibody that fits the normal colon mucosa stem cells, we screened cells using flow cytometry to examine colony formation, resistance to anticancer drugs, and tumor mass formation after subcutaneous implantation in mice.

The stem cell–containing region in the crypt of normal colon mucosa was negative for anti-Ki67 antibody staining; only the anti-CD44 v9 antibody stain was expressed. As for colony formation, resistance to anticancer drugs, and tumor mass formation, cells positive both for anti-CD44s and anti-CD44v9 antibody stains was significantly more frequent than those positive for anti-CD44s antibody stain and negative for anti-CD44v9 antibody stain and those negative both for anti-CD44s and anti-CD44v9 antibody stains.

CD44 variant exon 9 plays an important role in colon cancer stem cells.

## INTRODUCTION

The incidence of colon cancer is high among all malignant tumors[1,2]. In many cases, colon cancer spreads hematogenously to the liver and lungs. The elucidation of this mechanism may advance the development of new therapies and improve survival rates. To date, molecular biological research has been conducted on metastasis of colon cancer to the liver, and several molecular target drugs have been used in clinical settings[1,3].

In general, a stem cell in a normal tissue can develop into many constituent cells of a tissue and can remain alive continuously by structuring or restructuring the tissue[4]. A cancer stem cell(cancer initiating cell), likewise, is considered capable of self-replication, self-differentiation, drug resistance, and immune evasion[5-8]. In 1994, Dick and colleagues first identified stem cells in malignant tumors[9]. Using a molecular marker for human acute myeloid leukemia cells, they identified the cell that induces leuemia. In solid cancers, Michael Clarke found breast cancer stem cells in 2004[10]. Peter Dirks discovered cancer stem cells in brain tumors; [11] and O'Brien and Ricci-Vitiani reported the discovery of colon cancer stem cells in 2007[12,13]. All these reports showed that cancer stem cells play an important role in the differentiation and growth of cancer.

CD44, in particular, is a well-known marker for colon cancer stem cells. The gene, located on the short arm of chromosome 11, has a transmembrane structure, allowing at least 10 exons to be inserted into the extracellular domain near the transmembrane domain through an alternative splicing mechanism. A variant exon(v), which differs from the original exon, is inserted on the basis of tissue function[14]. It is involved in adhesion between cells and the extracellular matrix, cellular motion, and growth/invasion/metastasis of cancer cells[15,16]. However, the exon that plays the central role in cancer stem cells remains unknown. According to the recent report, hematogenous metastasis is likely to accompany the expression of the proteins inserted with CD44 variant exon 6 or CD44 variant exon 9(v9) in the primary foci of gastric and colon cancer cells[17-19]. According to a previous publication, breast cancer stem cells with CD44 protein expression are resistant to radiotherapy owing to an uncharacteristic rise of reactive oxygen levels in the cell[20]. A recent report has shown that the binding of CD44 variant exons (8, 9, and 10) to xCT proteins that transport cystine/glutamate on the cell membrane accelerates the formation of reduced glutathione and inhibits accumulation of reactive oxygen in cancer cells, thereby suppressing the activation of oxidative stress[21].

As discussed above, the CD44 molecule family is projected to be relevant at various levels of malignancy in tumors. In this study, we examined the stained images of stem cells from normal colon mucosa to explore the relationship between colon cancer stem cells and the CD44v9 isoform, which is considered important in this type of cancer.

## RESULTS

### Immunochemical staining of normal colon mucosa with anti-CD44s antibody and antibodies for each variant exon

**Figure 1:**
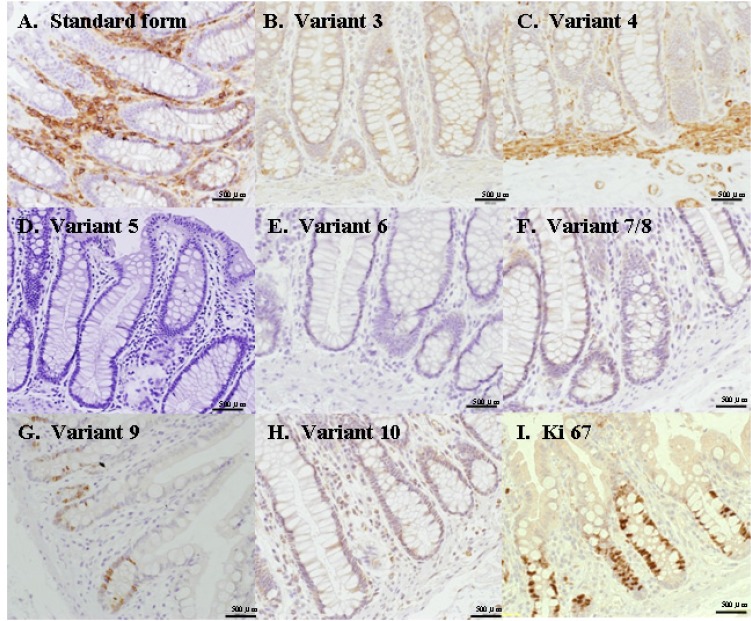
Immunohistochemical staining using anti-CD44 antibodies The expression was found with anti-CD44standard form(s) and anti-CD44 variant exon (3, 4, 5, 6, 7, 8, 9, and 10)antibodies. Among the anti-CD44 antibodies, only anti-CD44 variant exon 9(v9) induced expression in the anti-Ki67 antibody stain–negative region(stem cell).

Immunohistochemical staining was conducted using anti-Ki67 antibody, anti-CD44s antibody, and antibodies for each of the CD44 variant exons to examine the stem cells of normal colon mucosa(Fig. 1). Expression by anti-Ki67 antibody was found in transit-amplifying cells and differentiating cells in the normal mucosa, but expression was absent in stem cells in the crypt. Furthermore, in the anti-Ki67 antibody stain–negative region (stem cell), expression was found with anti-CD44s and anti-CD44 v3, v4, v9 and v10 antibodies. Among the anti-CD44 antibody group, only anti-CD44v9 induced expression in the anti-K67 antibody stain–negative region (stem cell).

### Cell separation via flow cytometry using anti-CD44s and anti-CD44v9 fluorescent antibodies

**Figure 2:**
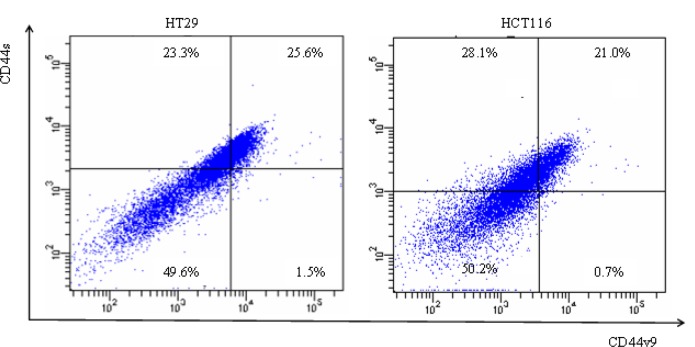
Flow cytometry of colon cancer cells using fluorescence anti-CD44s Ab and anti-CD44v9 Ab Flow cytometry was performed using colon cancer cell lines, HT29, HCT116 after they were stained for surface markers: CD44s, CD44v9. Vertical axis shows expression of CD44s, and horizontal axis showed expression of CD44v9. Three different sub populations were separated by fluorescence-activated cell sorting (FACS) as indicated: CD44s[+]v9[+], CD44s[+]v9[-], CD44s[-]v9[-].

After the reaction of anti-CD44s and anti-CD44v9 fluorescent antibodies with the colon cancer cell lines, fluorescence-activated cell sorting scanning was used to examine expression state (Fig. 2). The cells were separated into those that were expressed with both anti-CD44s and anti-CD44v9 antibodies(CD44s[+]v9[+]), those that were positive for anti-CD44s antibody but negative for anti-CD44v9 antibody(CD44s[+]v9[-]), and those negative both for anti-CD44s and anti-CD44v9 antibodies(CD44s[-]v9[-]).

### Colony formation in colon cancer cells expressed with anti-CD44v9 antibody

**Figure 3:**
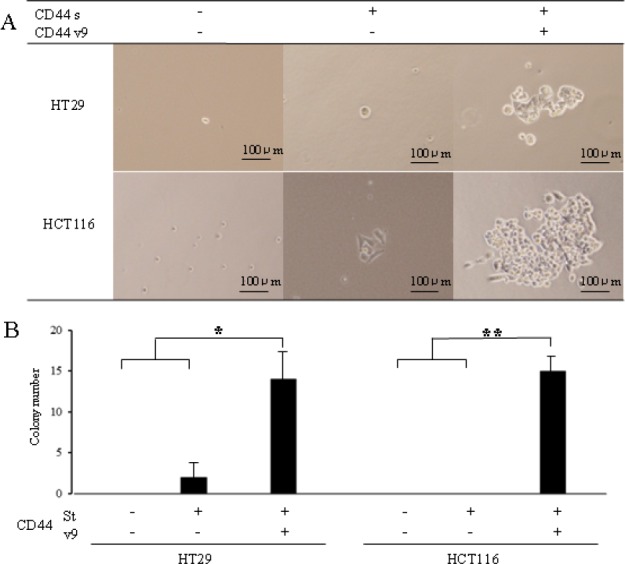
Colony formation in colon cancer cells expressed CD44s[+]v9[+], CD44s[+]v9[-], and CD44s[-]v9[-] A. Microscopic view of cells after 5 days. Colon cancer cells sorted as CD44s[+]v9[+], CD44s[+]v9[-], and CD44s[-]v9[-], separated 96-well plate as 1 cell per well using dilution limited technique. B. The number of colony formations. Results are presented as the mean±SD (n=4). P values were determined using the by Student's t-test. Values of < 0.05 were considered statistically significant. *p<0.05.**p<0.01.

After separation (see Fig 2), the cells were incubated in a 96-well plate (1 cell per well) for the investigation of colony formation. Figure showed a microscopic view of cells after five days(Fig. 3A). In HCT116 colon cancer cells, colony formation was absent in CD44s[-]v9[-], whereas only 2 colonies were formed by CD44s[+]v9[-]. Meanwhile, 14 colonies were formed by CD44s[+]v9[+] (Fig. 3B). In HT29 colon cancer cells, colony formation was absent in CD44s[-]v9[-] and CD44s[+]v9[-]. In anti-CD44s[+]v9[+], 145 colonies were formed. In both types of cancer cells, CD44s[+]v9[+] formed significantly more colonies than CD44s[-]v9[-] or CD44s[+]v9[-] did.(Fig. 3B)

### Resistance to 5-FU in anti-CD44v9 antibody–positive coloncancer cells

**Figure 4:**
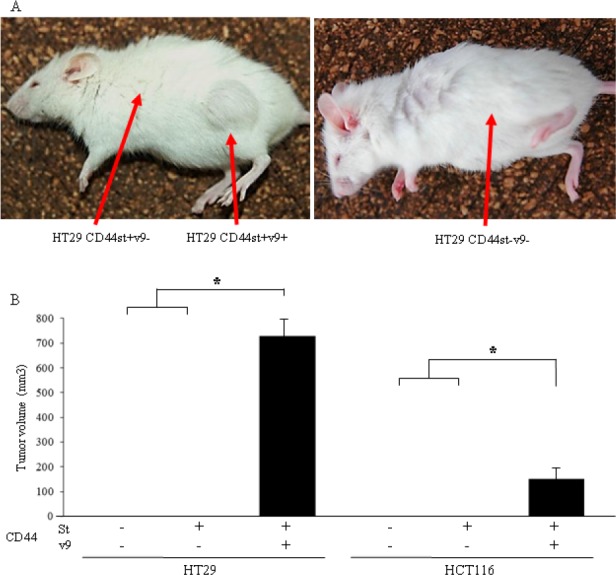
Colon cancer cells stained for ANNEXIN-V using by fluorescence microscope A.Photograph of cells stained for ANNEXIN-V. Colon cancer cells(1×10^4^) sorted to CD44s[+]v9[+], CD44s[+]v9[-], CD44s[-]v9[-] incubated in the presence of each concentration of 5-FU for 3 days. After incubating with annexin-V-Alexa568, we detected red cells under a fluorescent microscope. B. Result of living ratio against anticancer drug: 5-FU. After 72 hours of exposure, we detected non-red cells under a fluorescent microscope. Results are presented as the mean±SD (n=4). Left HT29 cells. Right HCT116 cells.

Screened colon cancer cell lines and the drug 5-FU were placed in a chamber. ANNEXIN-V staining was conducted to examine cell apoptosis and cell necrosis induction at various concentrations (Fig. 4A). In HT29 colon cancer cells, the non-staining rate of CD44s[+]v9[-] was 41.3% with 10 μg/mL, 17.5% with 30 μg/mL, and 14.7% with 50 μg/mL, whereas in CD44s[+]v9[+], the non-staining rate was 71.6% with 10 μg/mL and 48.7% with 50 μg/mL(Fig. 4B Left). In HCT116 colon cancer cells, the non-staining rate of CD44s[+]v9[-] was 64.6% with 10 μg/mL, and 22.5% with 50 μg/mL. The non-staining rate of CD44s[+]v9[+] was 87.5% with 10μg/mL, 81.6% with 30 μg/mL, and 67.8% with 50 μg/mL(Fig. 4B Right). In both colon cancer cells, the non-staining rate with ANEXIN-V in CD44s[+]v9[+] was significantly higher than that in CD44s[+]v9[-].

### Formation of tumor mass after subcutaneous implantation of anti-CD44v9 antibody–positive colon cancer cells in mice

**Figure 5:**
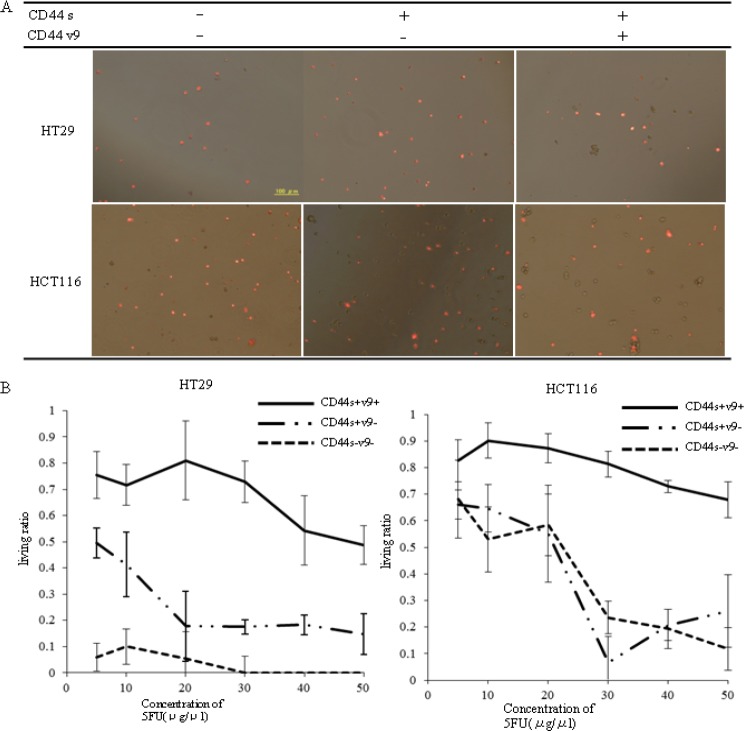
Evaluation of the tumorigenic potential of isolated CD44s and CD44v9 cells (A) Photograph of a mouse taken 4 weeks after HT29 cells transplantation. Each 1.0 × 10^3^ cells which sorted as CD44s[+]v9[+], CD44s[+]v9[-], CD44s[-]v9[-] were injected to NOD-SCID mouse subcutaneously. (B) Tumor volume of isolated CD44s and CD44v9 cells. The tumor size was measured with calipers, and calculated with the formula:(L × W^2^)/2(L; length of the tumor, W; width of the tumor). Results are presented as the mean±SD (n=4). P values were determined using the by Student's t-test. Values of < 0.05 were considered statistically significant.*p<0.01

No tumor mass was formed with CD44s[-]v9[-] or CD44s[+]v9[-] in HT29 colon cancer cells, although a 13×8×7-mm tumor mass was formed with CD44s[+]v9[+] (Fig. 5A). Also, in HCT116 colon cancer cells, no tumor mass was formed with CD44s[-]v9[-] or CD44s[+]v9[-], although a tumor mass was formed with CD44s[+]v9[+]. In both types of colon cancer cells, tumor masses formed only with CD44s[+]v9[+] (Fig. 5A,B).

## DISCUSSION

The concept of heterogeneity of cancer cells has recently undergone a major change. At the 2006 American Association for Cancer Research, cancer stem cells were defined as cells that “exist in a tumor, with capability of replicating themselves and generating different types of cancer cells that constitute the tumor tissue[25].” Based on this concept, a hierarchy model was proposed, in which highly oncogenic cancer cells and non-oncogenic cancer cells derived from oncogenic cells might coexist in a tumor tissue[26]. According to this model, the diversity and non-uniformity of cancer cells must be taken into account in planning therapy for full recovery.

The origin of this definition of cancer stem cells was the identification of blood stem cells by Till, MuCulloch, Becker, et al. in the 1960s, suggesting the involvement of stem cell-like cells in cancer growth[27]. In 2007, reports were published on stem cells for colorectal cancer, and CD44 was identified as a cancer stem cell marker. CD44, a transmembrane protein, may display extramembrane insertion of variant exons[28]. CD44 molecules with variant exon insertion have been found in blood cells, various normal tissues, and epithelial cancer cells. In the case of malignant tumors in particular, some variant exons are closely related to metastasis and malignancy, which indicates their importance[14-19]. However, no details about the expression of variant exons in CD44 or cancer stem cells in colon cancer are available.

Accordingly, we examined the expression of variant exons in CD44. First, assuming that stem cells in normal colon mucosa and those of colon cancer use the same exon, we conducted Ki-67 staining to identify the dormant cell at the bottom of the crypt, in which the stem cells of the normal colon mucosa are found. Next, we examined the expression of individual CD44 exons. According to the results, the expression in the Ki-67 stain–negative region was observed only with the vriant exon 9. The immunohistochemical staining of the colon cancer foci revealed that strong expression of the variant exon 9 was likely to cause hematogenous metastasis, and therefore, this exon was considered important[17].

By definition, cancer stem cells must have self-propagating or self-differentiating capability, as well as the capability to withstand the human immune system for survival. A cancer stem cell must also have resistance mechanisms against anticancer drugs and the capability to form tumor masses in immunodeficient mice(5-8). In this context, we compared CD44-positive colon cancer cells that did not contain the vriant exon 9 and those that did. CD44-positive colon cancer cells containing the variant exon 9 had significantly stronger colony–forming and tumor mass–forming abilities and resistance against anticancer drugs. Thus, in CD44 molecules in colon stem cancer cells, the variant exon 9 was considered an exceptionally important factor. Future studies on CD44 molecules and variant exon 9 are warranted for elucidating basic concepts of cancer propagation and drug resistance.

## MATERIALS AND METHODS

### Immunohistostaining

Normal colon tissues were obtained from a patient with primary colon cancer at the First Department of Surgery, University of Fukui, Japan, in 2005.

Surgical specimens of the adjacent normal colon tissues prepared from formalin-fixed, paraffin-embedded tissues were analyzed for protein expression by the ChemMate method using the EnVision system(DAKO, Danmark).

### Antibody(Ab)

The following antibodies were used: anti-human CD44 standard form(s) (Ab cam, UK), variant exon 3(Neuromics, MN, USA), 4(AbD serotec, UK), 5(AbD serotec), 6(R&D systems, MN, USA), 7/8(AbD serotec), 10(AbD serotec), anti-human CD44 variant exon 9(prepared in our department), and AE1/AE3(Santa Cruz Biotechnology, CA, USA), Ki-67(Invitorogen, CA,USA).

### Cell culture

The human coloncancer cell lines:HT29 and HCT116(Public Health England Culture Collection, UK) were cultured at 37C in 5% CO2 in RPMI 1640 medium(Sigma Chemical Co. MO, USA) containing 10% fetal bovine serum.[22]

### Flow cytometric analysis

Cells were washed twice with phosphate-buffered saline. Dissociated cells were stained with fluorescein isothiocyanate (FITC)-conjugated anti-CD44v9 antibody (prepared in our department)[19] and Allophycocyanin [APC]-conjugated CD44s antibody (BD Pharmingen, NJ, USA) and incubated for 30 minutes at 4°C. After washing with PBS, the cells were sorted by FACScan (Becton-Dickinson, CA, USA)

### Colony Formation Assay

Cells were plated at a density of 1 cell per well in 96 well plates (BD Falcon, USA). We examined the colonies under a microscope every 24 hours.

### Growth inhibitory assay

The cells were seeded in 96 well plates at 1 × 10^4^ cells/well and divided into control and treatment groups [5-Fluorouracl(5-FU) (100 μg/mM)] (Sigma Chemical Co.). After 72 hours of exposure, we detected non-red cells under a fluorescent microscope[23].

### Cell viability

Cell necrosis and apoptosis were detected by cytometry using Annexin-V-FLUOS staining Kit (Roche, Germany). Briefly, cells were incubated with annexin-V-Alexa568 for 15 minutes. After cells were washed thrice in PBS, we detected red and non-red cells under a fluorescent microscope.

### Tumorigenicity assays in nude mice

Six-week-old female NOD-SCID mice (Charles river, Japan) were subcutaneously injected in the right armpit region with 1.0 × 10^3^ cells in 0.1 mL of matrix gel(BD Biosciences, NJ, USA ). Three groups of mice were tested. Group 1 was injected with colon cancer cells positive both for anti-CD44s and anti-CD44v9 antibody stains(CD44s[+]v9[+]). Group 2 was injected with colon cancer cells positive for anti-CD44s antibody stain and negative for anti-CD44v9 antibody stain(CD44s[+]v9[-]). Group 3 was injected with colon cancer cells negative both for anti-CD44s and anti-CD44v9 antibody stains(CD44s[-]v9[-]). The tumor size was measured every 3 days with calipers. The tumor volume was calculated with the formula: (L × W^2^)/2, where L is the length and W is the width of the tumor[24].

### Statistical analyses

Differences between two groups were analyzed by Student's t-test. Values of P<0.05 were considered to indicate statistically significant results.
